# Targeting a cysteine protease from a pathobiont alleviates experimental arthritis

**DOI:** 10.1186/s13075-020-02205-z

**Published:** 2020-05-14

**Authors:** Hsin-Yi Peng, Shih-Yao Chen, Shih-Hong Siao, Jinghua Tsai Chang, Ting-Yin Xue, Yi-Hsuan Lee, Ming-Shiou Jan, Gregory J. Tsay, Moncef Zouali

**Affiliations:** 1 Division of Immunology and Rheumatology, China Medical University Hospital, Taichung, Taiwan; 2 Department of Internal Medicine, National Cheng Kung University Hospital, Tainan, Taiwan; 3 Graduate Institute of Immunology, National Taiwan University, Taipei, Taiwan; 4 Institute of Medicine, Chung Shan Medical University, Taichung, Taiwan; 5 College of Medicine, China Medical University, Taichung, Taiwan; 6 Institute of Biochemistry, Microbiology, Immunology, Chung Shan Medical University, Taichung, Taiwan; 7 Inserm UMR 1132, F-75475 Paris, France; 8 University Paris Diderot, Sorbonne Paris Cité, F-75475 Paris, France; 9 Graduate Institute of Biomedical Sciences, China Medical University, Taichung, Taiwan

**Keywords:** Rheumatoid arthritis, Periodontitis, *Porphyromonas gingivalis*, Gingipain, Collagen-induced arthritis

## Abstract

**Background:**

Several lines of evidence suggest that the pathobiont *Porphyromonas gingivalis* is involved in the development and/or progression of auto-inflammatory diseases. This bacterium produces cysteine proteases, such as gingipain RgpA, endowed with the potential to induce significant bone loss in model systems and in patients.

**Objective:**

We sought to gain further insight into the role of this pathobiont in rheumatoid arthritis (RA) and to identify novel therapeutic targets for auto-inflammatory diseases.

**Methods:**

We profiled the antibody response to RgPA-specific domains in patient sera. We also tested the potential protective effects of RgpA domains in an experimental arthritis model.

**Results:**

Pre-immunization of rats with purified recombinant RgpA domains alleviated arthritis in the joints of the rodents and reduced bone erosion. Using a functional genomics approach at both the mRNA and protein levels, we report that the pre-immunizations reduced arthritis severity by impacting a matrix metalloprotease characteristic of articular injury, a chemokine known to be involved in recruiting inflammatory cells, and three inflammatory cytokines. Finally, we identified an amino acid motif in the RgpA catalytic domain of *P. gingivalis* that shares sequence homology with type II collagen.

**Conclusion:**

We conclude that pre-immunization against gingipain domains can reduce the severity of experimentally induced arthritis. We suggest that targeting gingipain domains by pre-immunization, or, possibly, by small-molecule inhibitors, could reduce the potential of *P. gingivalis* to translocate to remote tissues and instigate and/or exacerbate pathology in RA, but also in other chronic inflammatory diseases.

## Key messages

- Pathobionts can translocate to remote tissues and instigate pathology.
- Pre-immunization against a cysteine protease from a pathobiont reduces disease severity.
- Targeting pathobiont could alleviate pathology in chronic inflammatory diseases.

## Introduction

Rheumatoid arthritis (RA) and periodontitis (PD) are two common human chronic inflammatory diseases. RA is the most frequent autoimmune disease, and its etiology remains unknown. With a significant prevalence worldwide, PD is an inflammatory disorder of oral tissues, including the gingiva, cementum, and periodontal ligament. For decades, associations between RA and PD have been noted, and mounting evidence suggests that PD-associated microbes could be involved in RA pathogenesis and that the presence of the pathobiont *Porphyromonas gingivalis* represents a risk in the development and/or progression of RA [1–3]. First, there is an increased prevalence of PD in RA patients, as compared to controls [4, 5]. Second, the presence of antibodies to *P. gingivalis* is associated with that of RA-related autoantibodies in subjects at increased risk for disease, but who have not yet developed RA [1]. Third, serum antibodies to *P. gingivalis* are present in significantly higher concentrations in patients with RA than in healthy controls, and their levels are in correlation with the presence of anti-citrullinated protein antibodies (ACPAs) that specify RA disease severity [1, 2]. Fourth, concentrations of circulating anti-*P. gingivalis* antibodies are associated with the expression of ACPAs detectable in the gingival crevicular fluid of PD patients [2, 6]. Fifth, citrullinated antigens that are present in the periodontium of PD patients are thought to play a role in triggering the immune response observed almost exclusively in RA [7]. Lastly, *P. gingivalis* DNA was found in the synovial fluid of patients with RA [8]. Yet the precise mechanisms underlying the interplay between the pathobiont, RA, and PD remain to be elucidated.

**Fig. 1:**
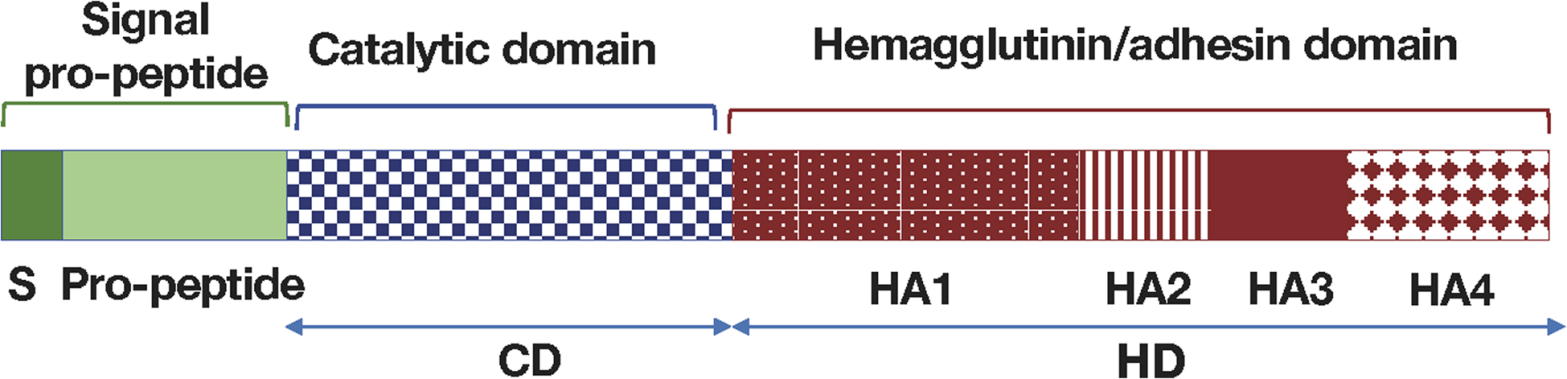
Protein domains of the RgpA gingipain complex. The catalytic, arginine-specific adhesin domain (CD) comprises 492 amino acids (positions 228–719). The hemagglutinin domain (positions 720–1703) is composed of 984 amino acids and comprises four sub-domains: HA1 (positions 720–1136), HA2 (positions 1137–1271), HA3 (positions 1272–1429), and HA4 (positions 1430–1703) [29]

*P. gingivalis* has evolved a cell surface-associated proteolytic system composed of several unique enzymes. In addition to participating indirectly in the pathological destruction of periodontal tissues, these enzymes also allow the bacterium to evade host defense mechanisms [9]. Among these virulence factors, cysteine proteases are endowed with a potential to deregulate the host immune response. Notably, *P. gingivalis* produces three cysteine proteinases known as gingipains: lysine-specific gingipain (Kgp), arginine-specific gingipain A (RgpA), and arginine-specific gingipain B (RgpB) [10–12]. They are essentially present on the outer membranes and outer membrane vesicles of virtually all *P. gingivalis* strains. The protein encoded by the *rgpa* gene is composed of a signal peptide, an N-terminal pro-fragment, an Arg-specific catalytic domain (CD), and a large C-terminal hemagglutinin/adhesin (HD) region (Fig. 1).

In animal models, oral challenge with *P. gingivalis* was demonstrated to stimulate bone loss [13]. Further investigation disclosed that gingipains produced by *P. gingivalis* have the potential to modulate the host immune response by affecting both the innate and adaptive branches of immunity, i.e., by degrading defensins [14], suppressing the cascade of complement activation [15], cleaving molecules expressed on T cells [16], proteolytically inactivating both anti-inflammatory (IL-4, IL-5) and pro-inflammatory (IL-12, IL-1b, IFN-g, and TNF-a) cytokines [17], and stimulating IL-6 production by oral epithelial cells [18] and IL-8 production by gingival fibroblasts [19], thereby hampering the protective immune response and enhancing inflammatory responses.

Experimental studies also revealed that the gingipains Kgp and Rgp exert distinct virulence roles. Immunization of mice with RgpA could stimulate the production of *P. gingivalis*- and RgpA-specific IgG antibodies, and the immunized animals against *P. gingivalis* or purified RgpA, but not against RgpB, were protected from maxillary bone loss [20]. Consistently, mice orally infected with either *P. gingivalis* wild type or gingipain mutants (*RgpA*^*−/−*^, *Kgp*^*−/−*^, *RgpA*^*−/−*^*/Kgp*^*−/−*^) exhibited a similar trend, and only RgpA-expressing bacteria showed significant bone loss and reduced phagocytosis, resulting in increased survival of *P. gingivalis* in the chamber exudates [21]. Together, the facts (i) that elimination of RgpA from *P. gingivalis* diminishes inflammation and augments phagocytosis and antibody titers, coincidental with reduced alveolar bone loss [21], and (ii) that only immunization of animals against purified RgpA, but not against RgpB, affords protection from bone loss [20] demonstrate that RgpA gingipain acts as a major virulence factor in pathogenesis.

Even though experimental evidence indicates that gingipains represent major virulence factors of *P. gingivalis*, the relative contributions of the catalytic and hemagglutinin domains of RgpA to pathogenesis in *P. gingivalis-*mediated human diseases remain unclear. To gain further insight into the potential roles of *P. gingivalis* gingipains in RA and PD, we therefore elected to focus on gingipain RgpA, known to be endowed with the potential to induce significant bone loss in model systems [20, 21]. Profiling of the antibody response to specific recombinant RgpA domains in patients with RA and PD led us to test the potential protective effects of RgpA domains in an experimental arthritis model.

## Materials and methods

### Study subjects

Serum samples were obtained from patients at Chung Shan Medical University Hospital (Taichung, Taiwan). All RA patients (*n* = 155) satisfied the American College of Rheumatology classification criteria [22]. PD subjects (*n* = 48) were selected from a pool of patients undergoing periodontal maintenance therapy (regular cleanings) for moderate to severe chronic PD. PD subjects were otherwise in good general health, and none of these subjects reported a diagnosis of RA. The Institutional Review Board of Chung Shan Medical University Hospital (Taichung, Taiwan) approved the studies. We also enrolled 35 healthy controls from a pool of available volunteers. They were matched to RA cases based on age and sex. Controls were selected on the basis of absence of any obvious joint or periodontal disease.

### Bacteria and culture conditions

*Porphyromonas gingivalis* (ATCC^R^ 33277™) was grown anaerobically at 37 °C in ATCC Medium (2722 Supplemented Tryptic Soy Broth/Agar) for 3 days. The bacteria were centrifuged at 6000*g* for 30 min at 4 °C. The cell pellet was used for injection to rats as described below.

### Cloning, expression, and purification of RgpA recombinant domains

The catalytic domain (CD), hemagglutinin domain (HD), and HA1, HA2–3, and HA4 sub-domains (Fig. 1) of *P. gingivalis* ATCC33277 were amplified from genomic DNA using forward and reverse primers containing the *EcoRI* and *XhoI* restriction sites and ligated into the yT&A vector (Yeastern Biotech Co. Ltd., Taipei, Taiwan). The plasmids were digested with *EcoRI* and *XhoI* and ligated into the expression vector pET32a(+) (Novagen, Madison, WI 53719) by DNA ligase (New England Biolabs, Ipswich, MA). The constructs were confirmed by nucleotide sequencing and expressed in *Escherichia coli* BL21-DE3 strain (Invitrogen). The bacteria were grown at 150 rpm in Luria–Bertani broth containing 100 μg/ml ampicillin at 37 °C in a shaking incubator. The transformed *E. coli* were grown until mid-log phase (OD = 1.0) by the addition of IPTG to a final concentration of 1 mM for approximately 14 h. After induction, the bacteria were centrifuged at 8000*g* for 10 min at 4 °C and stored at − 80 °C until use. The pellet (2 g) was resuspended in 40 ml of lysis buffer (12 mM sodium phosphate, 40 mM disodium phosphate, 300 mM sodium chloride, 6 M guanidine-HCl, 0.1 mM phenylmethylsulfonyl fluoride, pH 7.4) on ice, lysed by sonication, and centrifuged at 10,000 rpm for 10 min at 4 °C. For purification, TALON his-tag (Takara Bio USA, Inc.) was used. TALON is an immobilized metal affinity chromatography (IMAC) resin charged with cobalt, which binds to his-tagged proteins with high specificity, thereby delivering high purity his-tagged proteins. The bacterial supernatant was incubated with TALON resin for 5 min and washed three times with the equilibration buffer containing 5 mM imidazole. The bound protein was eluted with 150 mM imidazole. To remove imidazole and guanidine-HCl, a PD-10 Desalting Column (GE Healthcare Life Sciences, Marlborough, MA 01752) was used for desalting. Finally, the recombinant protein was quantified by the Bradford assay and analyzed by SDS-PAGE.

### Enzyme-linked immunosorbent assay (ELISA)

Antigens (CD, HA1, HA2–3, and HA4 domains of RgpA gingipain) were diluted in coating buffer (Protein Detector HRP Microwell Kit, KPL, USA). Wells of ELISA plates (Corning) were coated with 100 μl/well of protein solution (5 ng) or with coating buffer alone and incubated overnight at 4 °C. Wells were then blocked with 200 μl of PBS containing 2% BSA for 60 min at room temperature. Human sera were diluted 1/100 in blocking buffer, added in duplicate, and incubated overnight at 4 °C. After washing three times with 200 μl of PBS containing 0.1% of Tween 20, peroxidase-conjugated goat anti-human IgA or IgG antibody (KPL, Milford, MA) diluted 1/2500 in blocking buffer was added and incubated for 60 min at room temperature. After washing five times with 200 μl washing buffer, 100 μl of ABTS peroxidase substrate solution (KPL) was added. The reaction was stopped after 15 min by adding 100 μl of a peroxidase stop solution containing 5% sodium dodecyl sulfate. Absorbance was recorded at 405 nm by an ELISA reader (BioTek, Winooski, VT).

### Immunization of rats and induction of collagen-induced arthritis (CIA)

This study was approved by the Institutional Animal Care and Use Committee of National Cheng Kung University (Tainan, Taiwan). Thirty-six male Sprague-Dawley rats (6 to 7 weeks of age) were divided into six groups of six rats each. Rats received subcutaneous injections into 2 sites (one along the midline of the back between the shoulders, and the other on the lower back). Group 1 received 100 μl of PBS. Group 2 received 100 μl of incomplete Freund’s adjuvant (IFA). Group 3 received 100 μl of IFA containing 400 μg of heat-killed *P. gingivalis*. Group 4 received 100 μl of IFA containing 400 μg of hemagglutinin domain 4 (HA4). Group 5 received 100 μl of IFA containing 400 μg of the hemagglutinin domain (HD). Group 6 received 100 μl of IFA containing 400 μg of the catalytic domain (CD). Following these injections, CIA was induced in the pre-immunized animals [23]. For that purpose, each rat received an intradermal injection (0.5 ml) into four sites on the back containing 1:1 mixture of 400 μg bovine type II collagen (Elastin Products, Owensville, MO) dissolved in 0.1 M acetic acid and Freund’s complete adjuvant (Chondrex Inc., Redmond, WA 98052). Booster injections were administered after 7 days with 0.25 ml of the same emulsion into two different sites of the back.

### Assessment of joint arthritis

The articular index was calculated at 10, 12, 14, 16, and 18 days after CIA induction. For evaluation of arthritis severity, we used a previously described scoring system [24]: 0, no evidence of erythema and swelling; 1, erythema and mild swelling confined to the tarsals or ankle joint; 2, erythema and mild swelling extending from the ankle to the tarsals; 3, erythema and moderate swelling extending from the ankle to metatarsal joints; and 4, erythema and severe swelling encompass the ankle, foot and digits, or ankylosis of the limb. The incidence rate was calculated by counting the number of arthritic ankles relative to that of total ankles.

### Histopathological examination

Upon sacrifice on day 46, the ankles were removed, fixed in 10% formalin for 24 h, decalcified in formic acid for 7 days, and then embedded in paraffin, sectioned, and stained with hematoxylin and eosin (HE) or safranin O [23]. The HE-stained sections were scored for inflammation. The following scale was used: 0, no inflammation; 1, synovial inflammation, mild infiltration; 2, mild synovial inflammation; 3, moderate synovial inflammation; and 4, synovium highly infiltrated with many inflammatory cells. Safranin O staining was scored for cartilage damage with the following scale: 0, no destruction; 1, slight reduction; 2, moderate reduction; 3, severe reduction; and 4, absence of staining.

### Measurement of inflammatory markers in the serum and joints of the rats

The levels of serum matrix metalloproteinase-9 (MMP-9) (Biolegend, San Diego, CA), IL-1β (Biolegend), TNF-α and IL-17 (Life Technologies, Carlsbad, CA), and CXCL-1 (Abcam, Cambridge, UK) were measured by ELISA using commercially available kits according to the instruction of the manufacturers. Expression of MMP-9, IL-1β, TNF-α, and IL-17 was also measured by quantitative real-time PCR. To that end, total RNA was isolated from rat synovial tissue and extracted using TRIzol Reagent (Life Technologies, Carlsbad, CA). For cDNA synthesis, we used iScript cDNA Synthesis Kits (BioRad, Hercules, CA). Real-time PCR was performed using Q SYBR Green Supermix (BioRad). The following primers were used: (1) MMP-9 (forward: GGATGGTTATCGCTGGTG; reverse: AGTAGGACAGAAGCCATACA), (2) IL-1β (forward: TTCGACAGTGAGGAGAATGACC; reverse: CAAGACATAGGTAGCTGCCACA), (3) TNF-α (forward: CCTCACACTCAGATCATCTTCTCA; reverse: CTCCTCCGCTTGGTGGTT), (4) CXCL-1 (forward: ACCGAAGTCATAGCCACACTC;, reverse: CGCCATCGGTGCAATCTATCT), and (5) IL-17 (forward: CATCCATGTGCCTGATGCTGTTG; reverse: GGAACGGTTGAGGTAGTCTGAGG). The data were normalized to the expression levels of β-actin (forward primer: CAACGGCTCCGGCATGTGC, reverse primer: CTCTTGCTCTGGGCCTCG). The PCR conditions were 40 cycles at 95 °C for 30 s, 60 °C for 30 s, and 72 °C for 30 s.

### Probing expression of IL-17 in the joints of the rats by immunohistochemistry

For detection of IL-17 in situ, we used the UltraVision™ Quanto Detection System HRP DAB (Thermo Fisher Scientific, Waltham, MA) according to the instruction of the manufacturer. Briefly, tissue sections from three rats per group were deparaffined and rehydrated. To reduce nonspecific background staining due to endogenous peroxidase, slides were incubated with Hydrogen Peroxide Block or 10 min. After washing, sections were incubated with Ultra V Block for 5 min to block nonspecific background staining. Slides were then incubated overnight at 4 °C with an anti-IL-17 primary antibody (1 μg/ml, GeneTex, Irvinne, CA) diluted in citrate buffer pH 6. After three washes, binding was revealed by adding DAB Quanto Chromogen diluted DAB Quanto substrate for 5 min. IL-17 staining was quantitated using the Aperio ImageScope viewing software (Leica Biosystems, Wetzlar, Germany).

### Statistical analysis

All data were expressed as mean ± standard error of the mean (SEM). Statistical analysis was performed using GraphPad Prism v6.01 software. The Mann–Whitney *U* test was used to test statistical significance, and a *p* value < 0.05 was considered statistically significant.

## Results

### Cloning and expression of gingipain domains

In the present study, a total of four gingipain domains of *P. gingivalis* origin were targeted: the arginine-specific catalytic domain (CD) and components of the hemagglutinin/adhesin domain (HA1, HA2–3, and HA4). The molecular weights of the respective proteins are as follows: CD = 58 kDa, HA1 = 72 kDa, HA2–3 = 36 kDa, and HA4 = 53 kDa (Fig. 1). The genes encoding these four *P. gingivalis* proteins were cloned, and the recombinant proteins produced in appropriate expression vectors, as described in the “Materials and methods” section. Western chemiluminescence analysis of these proteins blotted with an anti-histidine-tagged antibody revealed protein bands exhibiting the expected molecular weights (Supplementary Figure 1). These proteins were then used for screening the serum immune-reactivity of patients with RA and PD. Sera from control individuals were used as controls.

### Profiling serum antibody reactivities with gingipain domains in PD and RA

**Fig. 2:**
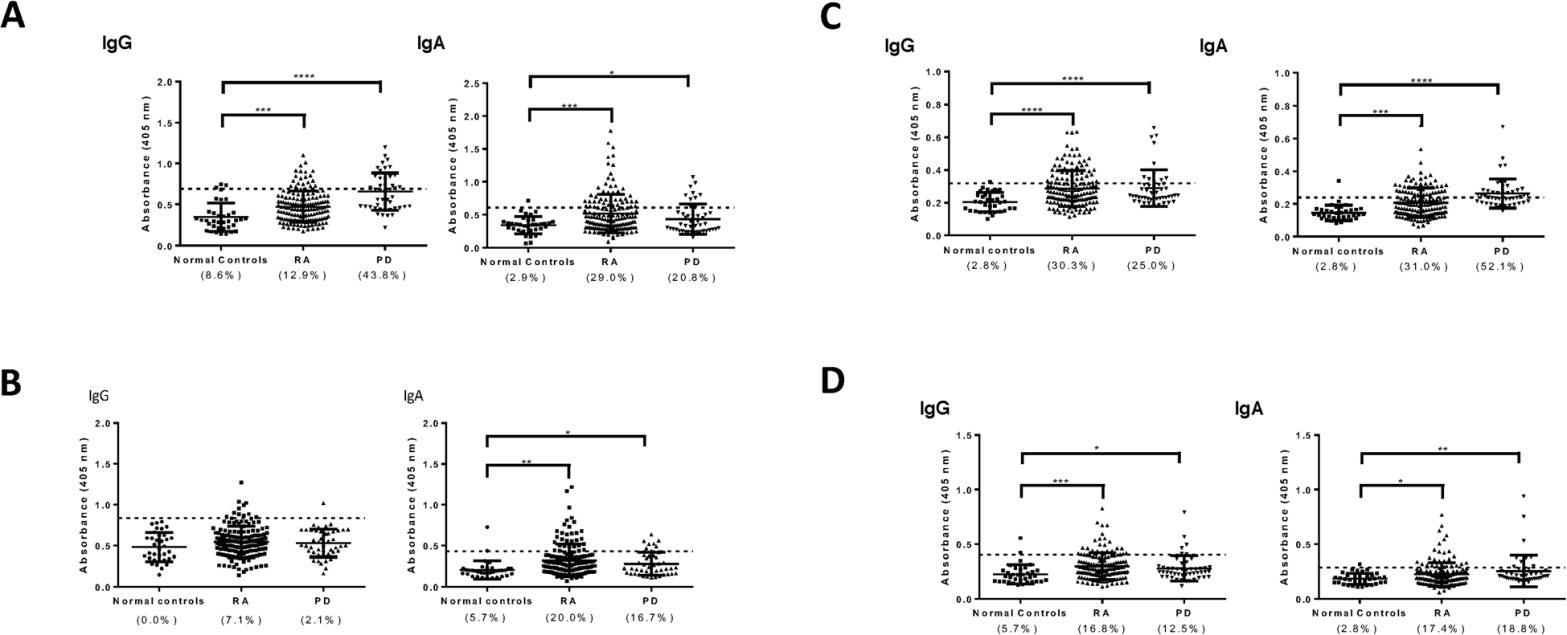
Immune-reactivity of serum human antibodies with gingipain domains. Sera from patients with RA (*n* = 155) or with PD (*n* = 48) were tested for reactivity with recombinant proteins corresponding to the HA1 (**a**), HA2/3 (**b**), HA4 (**c**), and CD (**d**) gingipain domains of *P. gingivalis* by ELISA. Sera from normal subjects (*n* = 35) were used as controls. Antibodies of the IgG isotype are shown on the left, and IgA antibodies on the right side of each panel. Values above the mean + SD were considered significant. *p < 0.05; **p < 0.05; ***p < 0.001; and ****p < 0.0001

A potent serum IgG response to the HA1 and HA4 gingipain domains was present in patients with periodontitis (Fig. 2). We also found that IgA antibodies to HA1 and HA4 are detectable in approximately 50% of the patients. In addition to the HA1 and HA4 gingipain domains, PD patients had elevated levels of IgG and IgA antibodies to the HA2/3 and CD domains, as compared to control subjects.

We next profiled sera from RA patients for the presence of elevated levels of IgG and IgA antibodies to gingipain domains using an ELISA assay. The titers of antibodies of the two isotypes were significantly increased in RA patients, as compared to control subjects (Fig. 2). For the HA4 and CD domains, the prevalence of IgG antibodies was even higher in RA than in PD. Importantly, sera from patients with the autoimmune disease SLE did not exhibit increased levels of antibodies to gingipain domains, indicating that the antibody reactivities detected were not the result of nonspecific binding (Supplemental Figure 2).

We then sought to confirm the ELISA reactivities by Western blotting. Serum samples from five control subjects that scored negative in the ELISA assays were found negative for HA1, HA4, and CD gingipain domains, indicating a strong positive correlation between the ELISA and the Western blotting data (Supplemental Figure 3). Five sera from RA patients and five sera from PD patients were also screened by Western blotting. There was generally a strong correlation between ELISA and Western positivity. For RA sera, there was a positive correlation between anti-HA4 reactivity levels measured by ELISA and band intensity revealed by Western, and three samples that scored negative in ELISA were positive for HA1 by Western blotting.

We conclude that both PD and RA patients exhibit high levels of IgG and IgA antibodies to the different gingipain domains we have tested. The prevalence of increased levels of antibodies and their intensity were higher for the HA domains than for the CD domain. Strikingly, the prevalence of increased levels of IgG to HA2–3, HA4, and CD domains was higher in RA than in periodontal disease. However, disease severity was not in correlation with antibody levels against RgpA gingipain (Supplementary Figure 4).

### Evolution of experimental arthritis following pre-immunization with gingipain domains

**Fig. 3:**
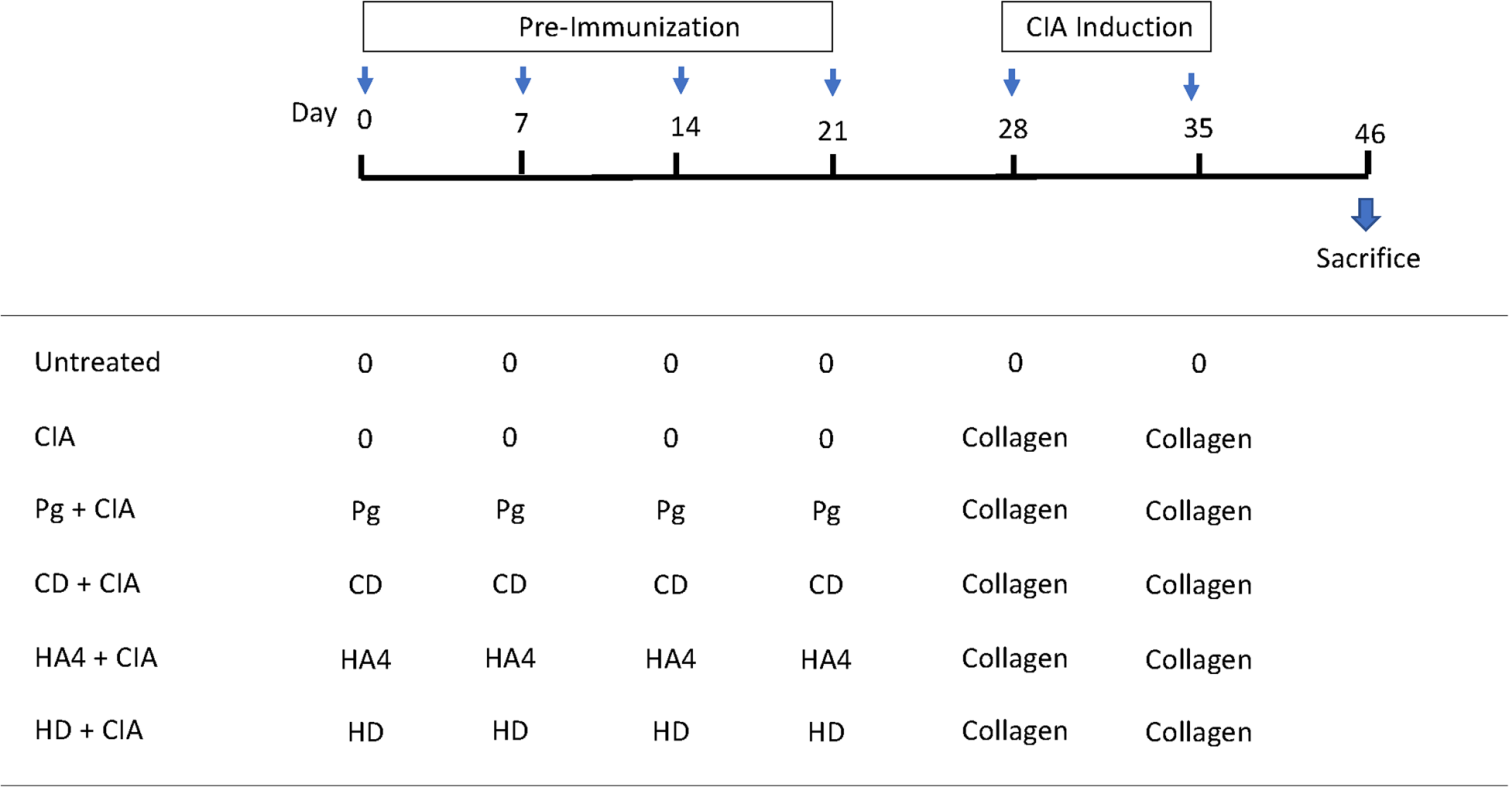
Schematic representation of the in vivo experimental design. Rats were pre-immunized with the indicated antigens for 21 days, followed by induction of CIA by two consecutive intradermal inoculations of bovine type II collagen. Rats were monitored regularly for visible signs of arthritis and were sacrificed at day 46. The time scale is shown in days. Sera were collected at sacrifice. Pg, heat-inactivated *Porphyromonas gingivalis*; HA4, recombinant fourth domain of the hemagglutinin/adhesin domain of RgpA; HD, recombinant hemagglmutinin/adesin domain of the arginine-specific cysteine proteinase RgpA; CD, recombinant catalytic domain of RPG; CIA, collagen-induced arthritis

We assessed the effect of pre-immunization against several gingipain domains on CIA development. To that extent, rats were first immunized with either the whole pathobiont or purified recombinant proteins. CIA induction was then induced by injecting type II collagen as depicted in the experimental diagram (Fig. 3). All groups were followed for identical time periods. Rats that received *P. gingivalis*-purified antigens showed elevated antibody titers to CD, HD, and HA4 recombinant proteins, which was considered proof of successful immunizations (Supplementary Figure 5).

**Fig. 4:**
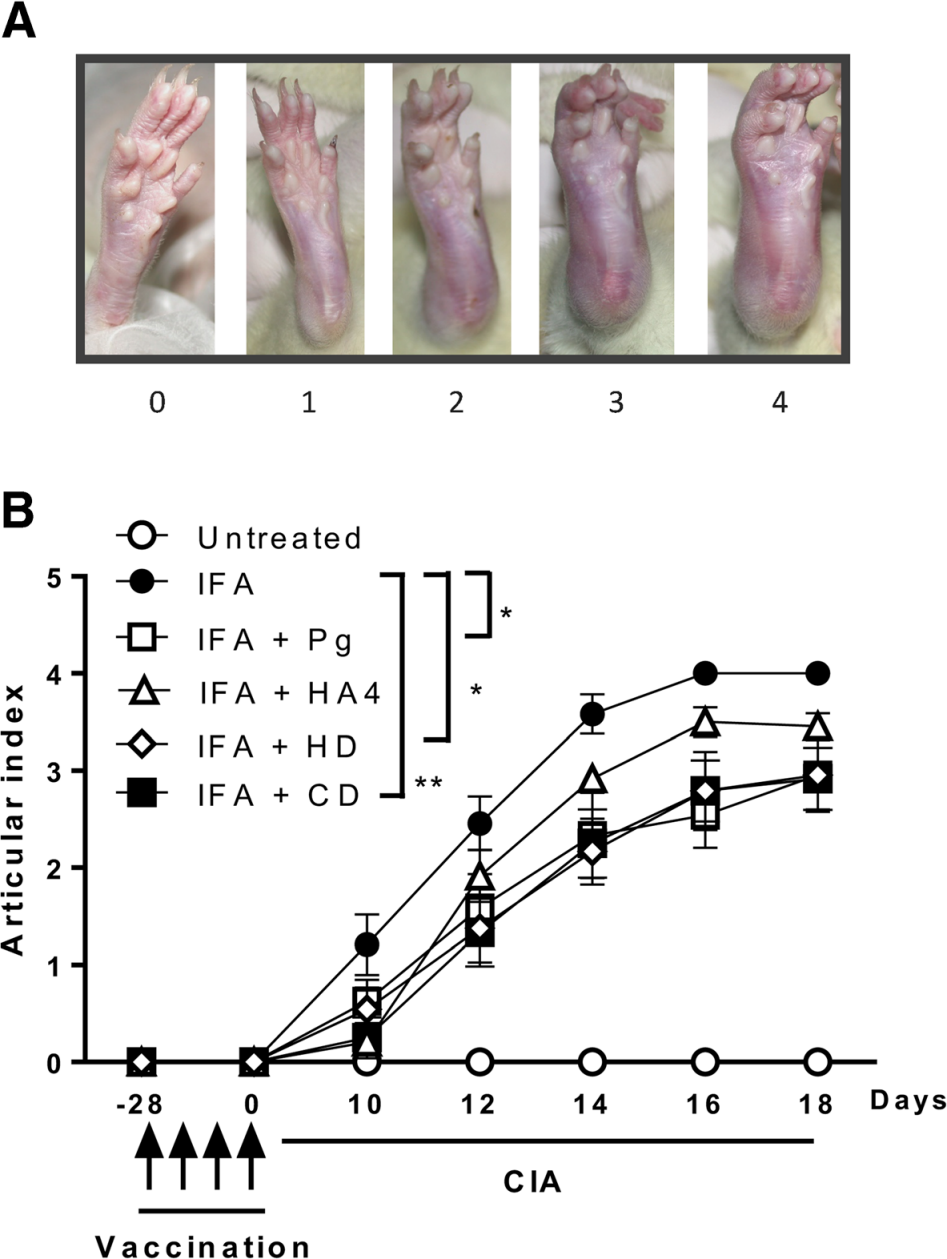
Evolution of experimental arthritis following pre-immunization with gingipain domains. **a** Scoring of the articular index in rats immunized with type II collagen. The scores used were on a 0–4 scale, as described in the “Materials and methods” section [24]. Shown are representative views of the ankles as the severity of the arthritis progresses: 0, no swelling or erythema; 1, slight swelling and/or erythema; 2, low to moderate edema; 3, pronounced edema with limited joint usage; and 4, excess edema with joint rigidity. **b** Effects of pre-immunization with recombinant gingipain domains on the severity of collagen-induced arthritis (CIA). Thirty-six rats were divided into six groups and received subcutaneous injection of PBS, incomplete Freund’s adjuvant (IFA), emulsified heat-killed *Porphyromonas gingivalis* (Pg), and emulsified HD, CD, and H4 domains of RgpA every week for 28 days. CIA was then induced by intradermal injections of bovine type II collagen. The clinical severity of arthritis in each paw was quantified throughout the experimental period according to a graded scale from 0 to 4. The scores for all four paws were summed to generate a representative arthritis score. Results are presented as mean ± SEM. *p < 0.05; **p < 0.01

Scoring of the articular index in rats immunized with type II collagen is shown in Fig. 4a, and the time-course of macroscopic score values and arthritis incidence are summarized in Fig. 4b. Rats that only received collagen developed arthritis beginning at day 10 and inflammation increased progressively with time. We noted that rats that were pre-immunized with *P. gingivalis* or gingipain fragments HA4 or CD before CIA induction had reduced arthritic score values. Eighteen days after CIA induction, rats that had been pre-immunized with CD had the lowest articular index (2.92 ± 1.10, *p* < 0.01) compared to rats suffering from arthritis (4.00 ± 0.00). At the end of the observation period, on day 46, rats were sacrificed, and joints and sera were collected for further analysis.

### Histopathological analysis of arthritic joints from CIA rats

**Fig. 5:**
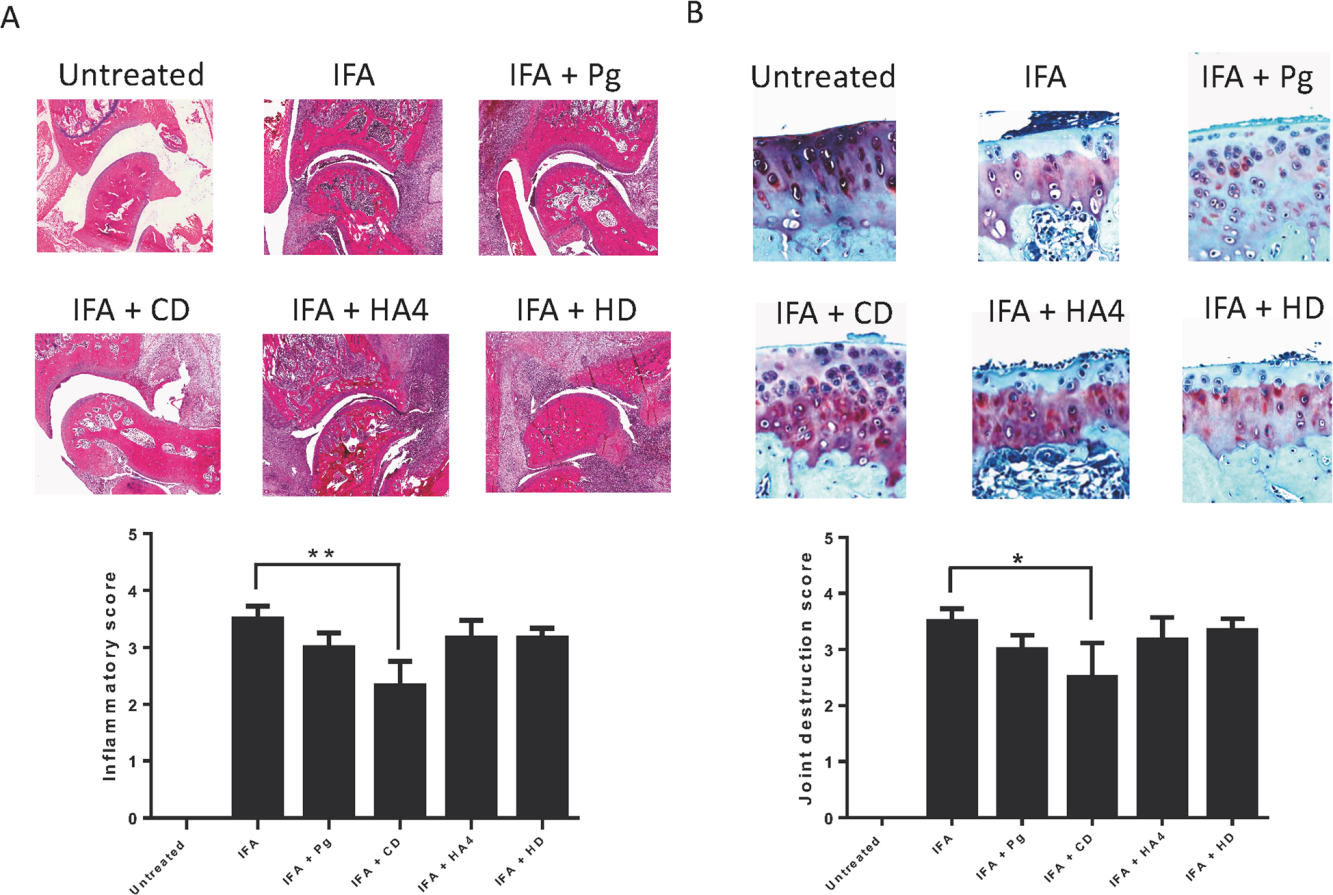
Histopathological analysis of ankle joints from CIA rats pre-immunized with recombinant gingipain domains. Tissue sections of joints in paws of animals from each group were stained with hematoxylin and eosin (**a**), or safranin O and toluidine blue (**b**). Inflammation cores (**a**) and joint destruction scores (**b**) were calculated for each group. In **a**, the following scale was used: 0, no inflammation; 1, synovial inflammation, mild infiltration; 2, mild synovial inflammation; 3, moderate synovial inflammation; and 4, synovium highly infiltrated with many inflammatory cells. In **b**, safranin O staining was scored for cartilage damage with the following scale: 0, no destruction; 1, slight reduction; 2, moderate reduction; 3, sever reduction; and 4, absence of staining. Shown are representative histological sections of ankle joints. The untreated control group displayed normal, healthy articular space and tissues. In control rats that were not pre-exposed to RgpA proteins, synovial hyperplasia, destruction of articular cartilage, and close articular cavity are manifestations of CIA. In the group pre-immunized with CD, there was reduction in histopathological manifestations, including synovial hyperplasia, destruction of articular cartilage, and articular cavity changes compared with the CIA control group. The results are presented as mean ± SEM. *p < 0.05; **p < 0.01

Since inflammation in the joints of CIA rats immunized with gingipain fragments was reduced, we performed hematoxylin and eosin staining to assess ankle joint histopathology in the different test groups. As expected, the untreated group exhibited no detectable inflammation or tissue destruction in the joints of the rats. Compared with the untreated control group, the joints of the CIA rats exhibited significant pathological changes, synovial hyperplasia, infiltration by inflammatory cells, and disorderly cell arrangements typical of arthritis (Fig. 5a). We noted that rats that had been pre-immunized with gingipain fragment CD had reduced inflammatory scores, compared to un-immunized rats. Importantly, pre-immunization with the CD domain before CIA induction imparted the highest beneficial effect to the rats and resulted in a significant decrease of the inflammatory scores and reduction in inflammatory cells infiltrating the synovium, as compared to control rats (2.33 ± 1.02; *p* < 0.01).

To determine if the reduction in inflammatory cell infiltration and joint destruction also is associated with cartilage changes, we performed safranin O and toluidine blue staining to probe the cartilage layers and to assess the degradation and destruction of cartilage tissue. In control rats, the cartilage was intact and large amounts of safranin O staining were observed. Rats suffering from CIA exhibited cartilage thinning and erosion in the joints (Fig. 5b). Remarkably, and consistent with the observations made with hematoxylin and eosin staining, rats that had been pre-immunized with gingipain fragment CD had less severe cartilage erosive changes and the damage scores were significantly reduced, as compared to control rats (2.50 ± 1.51, *p* < 0.05). Thus, the macroscopic overt signs of arthritis seen in the rats were paralleled by histopathological markers of inflammation and joint destructions.

### Impact of the pre-treatments on mediators of inflammation and bone erosion

**Fig. 6:**
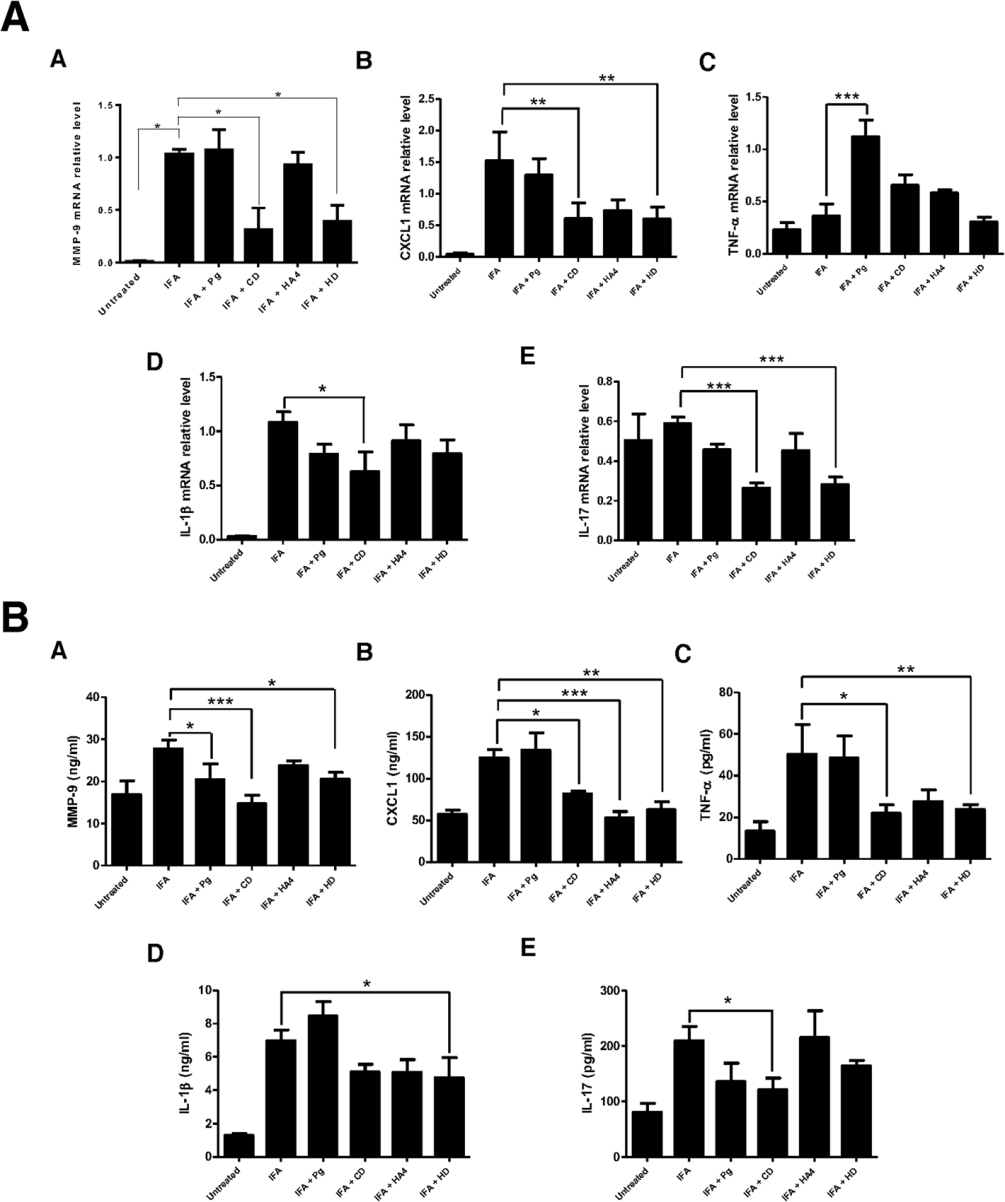
Impact of the pre-treatments on mediators of inflammation and bone erosion. **a** Effects of pre-immunization with RgpA domains on mRNA expression levels of inflammatory markers in joint tissue of CIA rats. Real-time PCR was used to quantify expression of MMP9, CXCL1, TNF-alpha, IL-1b, and IL-17 in the ankle synovial tissues of rats. Expression levels were quantified by densitometric analysis of bands and normalized to those of GAPDH mRNA. Data are recorded as mean ± standard deviation from six animals per group. Results are presented as mean ± SEM. *p < 0.05; **p < 0.01; and ***p < 0.001. **b** Effects of pre-immunization with RgpA domains on serum levels of inflammatory markers in CIA rats. Levels of MMP9, CXCL1, TNF-alpha, IL-1b, and IL-17 were assessed in the sera of the different groups. Results are presented as mean ± SEM. *p < 0.05; **p < 0.01; ***p < 0.001

Among the seven matrix metalloproteinases that are expressed under varying circumstances in the articular cartilage, the expression of MMP-9 in the cartilage is considered to be characteristic of articular injury [25]. Here, we found that pre-treatment of rats with gingipain fragments CD before CIA induction significantly reduced expression of mRNA MMP-9 levels (Fig. 6a). This reduction was also detectable at the protein level wherein CD pre-treatment had the most significant effect (*p* < 0.001, Fig. 6b).

In RA, chemokines play a role as mediators that can recruit inflammatory cells, and they are abundantly expressed in the serum and synovial tissues of patients. In rodents, CXCL-1 has been reported to mediate neutrophil recruitment to the joints of arthritic mice, and its upregulation in ankle and synovial fluid parallels disease progression [26]. Similar to what we observed for MMP9, we found that gingipain fragments, particularly CD, decreased CXCL-1 levels at both the mRNA and protein levels (Fig. 6).

During erosive arthritis, inflammatory cytokines such as IL-1 beta, TNF-alpha, and IL-17A trigger the production of enzymes, including MMP9, that can degrade components of the extracellular matrix. In the present studies, gingipain fragment CD had an impact on expression of these three cytokines locally in the joints, or systemically in the serum (Fig. 6).

**Fig. 7:**
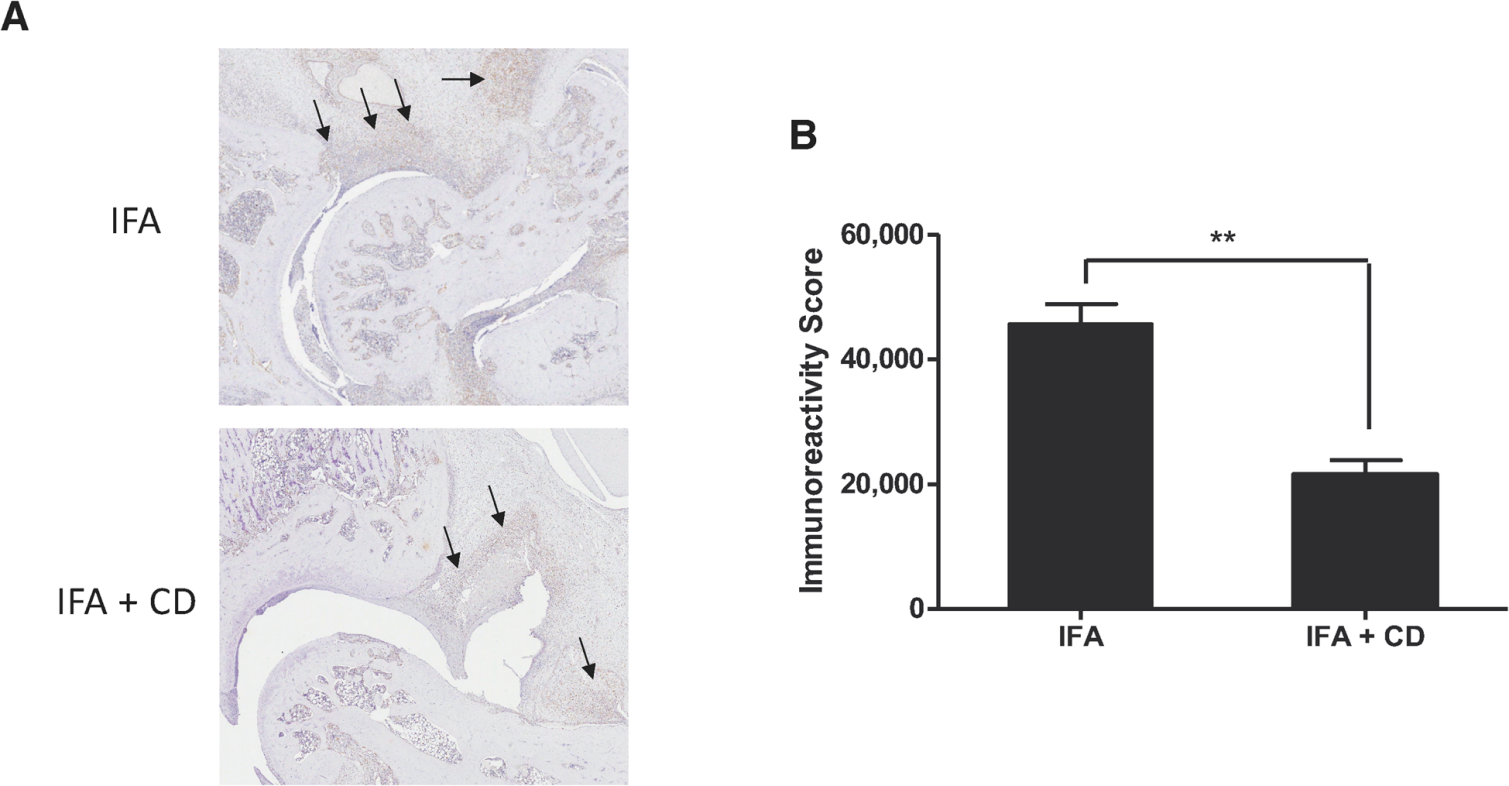
Reduced IL-17 expression in the synovial tissue of CIA rats by pre-immunization with the CD domain of RgpA. Briefly, joint tissue sections from three rats per group were deparaffined and IL-17 expression was assessed by immunohistochemistry as described in the “Materials and methods” section. Synovial tissue is denoted by arrows. Results are presented as mean ± SEM. **p < 0.01

IL-17A directly contributes to early induction and late chronic stages of RA, where this cytokine induces synovial changes that result in synovitis and sustain local inflammation [27]. Here, we found that pre-treatment of rats with gingipain fragment CD before CIA induction markedly reduced the expression of mRNA IL-17 levels in the synovium and IL-17 protein levels in the serum (Fig. 6). Given the prominent role of this pro-inflammatory cytokine in both human and experimental arthritis, we also probed its expression in the synovium of rats by immunohistochemistry. The results showed that CD pre-treatment significantly reduced local IL-17 expression in rats suffering from arthritis (*p* < 0.01, Fig. 7).

### A motif of the RgpA catalytic domain shares sequence homology with type II collagen

**Fig. 8:**
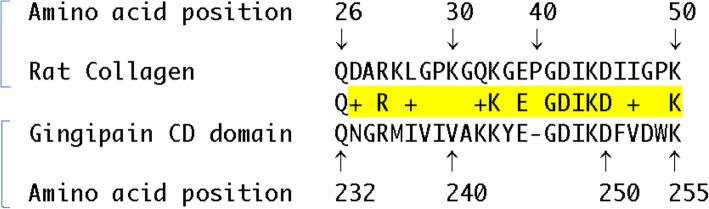
An amino acid motif shared by the catalytic domain of gingipain R and rat type II collagen. Shown are the sequences of amino acids common to the CD domain of gingipain and rat type II collagen. The UniProt numbering was used for gingipain (entry code B2RM93) and for the gene Col2a1 encoding collagen alpha-1(II) chain of *Rattus norvegicus* (entry code P05539)

The crystal structure of gingipain has been resolved at 1.5-Å resolution [28]. It revealed that the CD lies in a crown formed by the N-terminal 351 residue and exhibits the structural motif of a typical α/β protein. The active-site region includes several important residues, including D163, H211, V242, A243, C244, Y283, and Y325 [28]. We performed searches for amino sequence homologies between gingipain and rat type II collagen. We identified a motif of 25 residues that includes 10 identical amino acids (Q232, R235, K243, E245, G246, D247, I248, K249, D250, and K256) and four sets of residues that exhibit similar charge and/or side chain properties, i.e., D/N, Q/K, and L/I, V/I (Fig. 8). Since this amino acid motif (residues 232 to 256) overlaps with the active site of the catalytic domain (residues 163 to 325) of gingipain [28], it must be exposed at the protein surface. Because solvent-exposed amino acids are known to be immunogenic, it is plausible to propose that this motif is able to trigger a potent protective immune response in the pre-immunized CIA rats.

## Discussion

Converging observations indicate that there are tight connections between RA and PD. To gain insight into the mechanistic links between these disorders and to clarify the consequences of exposure to *P. gingivalis* on RA progression, we profiled the antibody response of patients with RA and PD to specific domains of gingipain RgpA. In line with other observations [29, 30], we found that both PD and RA patients exhibit high levels of IgG and IgA antibodies to the different gingipain domains we have tested. Notably, the prevalence of increased levels of antibodies and their intensity were higher for the HD than for the CD domain. This finding can be explained by the fact that the CD contains post-translationally added carbohydrates that could act as a shield to evade immune recognition in subjects who have encountered *P. gingivalis* [31]. Consistent with this view, the recombinant CD domain we have used was expressed in a prokaryotic system and is, therefore, likely to exhibit a set of epitopes distinct from those expressed by the native CD to which the patients have been exposed. Thus, the lower immunogenicity of the CD in hosts who have previously encountered *P. gingivalis*, together with the use of an unglycosylated form of CD in immunoassays are likely to account for the low human serum antibody response to this domain, as compared to that directed to HD domains.

The distinct profiles of antibody responses to the RgpA domains in RA patients prompted us to test the potential effects of pre-immunization against these proteins on experimental arthritis. Strikingly, we found that recombinant domains devoid of post-translation glycosylation had beneficial effects on arthritis development in the rat. Naturally, CIA is only an experimental model, and the results obtained herein cannot be directly translated to the human disease. Nevertheless, our observations confirm the existence of a link between the mucosa and the joint, as has been reported by other groups. Our findings also revealed, for the first time to our knowledge, the benefit of immunization against *P. gingivalis*-specific domains in alleviating experimental injury of the joint.

In assessing inflammatory markers in the serum and joints of CIA rats, we considered the fact that mRNA expression levels are not always faithful indicators of protein expression, particularly when comparing different genes [32]. First, complex biological processes, i.e., transcriptional and post-transcriptional splicing, translational regulations, and protein complex formation, can affect differently the relative quantities of mRNA and protein of the genes tested, especially for enzymes with structural changes at the catalytic core, such as MMP-9. Second, distinct mRNA and protein degradation rates can impact the mRNA and protein correlations in different cell types. Third, different mRNA secondary structures can give rise to different protein translation efficiencies. Fourth, some mRNAs can be transcribed, but not translated, implying that the number of functional protein molecules is not necessarily a reflection of the number of mRNA copies. Finally, whereas the degradation rate of mammalian mRNAs falls within a tight range of 2 to 7 h, the half-life of different proteins can vary from minutes to days. Therefore, we used a functional genomics approach at both the mRNA and protein levels to probe the inflammatory pathways impacted by the immunizations, i.e., MMP-9, CXCL1, TNF-alpha, IL-1beta, and IL-17A. These two complementary and informative expression levels enabled us to quantify several biomarkers of rat arthritis. Overall, gene expression at the mRNA level was correlated with that of the protein expressed. We therefore conclude that the pre-immunizations used reduced arthritis severity by impacting a matrix metalloprotease characteristic of articular injury, a chemokine known to be involved in recruiting inflammatory cells, and three inflammatory cytokines.

As is the case for the human disease, production of arthritogenic antibodies and autoreactive T cells are hallmarks of the rat CIA model [33]. In the rat, the antibodies produced cross-react with the animal’s own collagen, fix the complement, and bind to cartilage in the joint of the immunized rodents [34]. Intravenous injection of a human anti-type II collagen antibody to mice induced synovitis, indicating that antibodies to collagen can play a potent pathogenic role [34]. In an attempt to decipher the mechanisms underlying the beneficial effects of pre-immunization with Rgp domains on CIA, we identified an amino acid sequence homology between the CD domain and rat type II collagen. Since the motif we have identified overlaps with the active catalytic site, is solvent-exposed on gingipain, and, hence, potentially immunogenic, it is conceivable that pre-immunization of rats against CD gave rise to type II collagen-specific antibodies that alleviated arthritis in the joints of the animals by preventing anti-collagen antibodies from inducing tissue lesions.

Investigation of whether pre-exposure of animals to *P. gingivalis* can affect the progression of experimental arthritis is not without precedent. In one study, mice immunized with type II collagen received five oral inoculations of *P. gingivalis* every other day [35]. This regimen induced alveolar bone loss typical of periodontitis, but also accelerated arthritis onset. In another study, the SKG mouse model was used [36]. This strain exhibits a single point mutation in the zeta-chain-associated protein kinase 70 (ZAP-70) gene that results in abnormal T cell selection. Following induction of arthritis by laminarin derived from a brown alga called *Laminaria digitate*, injection of *P. gingivalis* exacerbated experimental arthritis [36]. These two studies, suggesting that administration of *P. gingivalis* can exacerbate RA in mice, differ from our current observations for several reasons. First, whereas in the first study [35], mice were gavaged with *P. gingivalis*, we have used the intraperitoneal route. Second, the significance of the SKG studies [36] is unclear because this mouse strain exhibits a gene defect that is not present in the model we have used, nor in RA patients. Third, while the two studies [35, 36] made use of whole *P. gingivalis* bacteria, we further used purified recombinant proteins corresponding to different gingipain domains. Bacterial components of *P. gingivalis*, such as lipopolysaccharides (LPS), are known to target toll-like receptors, including TLR-2 and TLR-4, which can affect osteoclastogenesis. It is, therefore, conceivable that the exacerbations of arthritis reported elsewhere [35, 36] were the result of confounding inflammatory effects of LPS derived from *P. gingivalis*.

Importantly, most recent studies of the effects of pre-exposure to *P. gingivalis* on bone erosion support the conclusions of our present investigations. First, immunization of mice with a combination of two RgpA recombinant domains (HA1 and HA2) led to protein-specific antibodies that proved to be protective from *P.* gingivalis-elicited oral bone loss [37]. Second, investigators used a mouse model wherein PD was provoked by oral gavage with *P. gingivalis* [38]. CIA was then induced by injecting type II collagen, and mice were subjected to adjunctive anti-microbial therapy or systemic antibiotics. Remarkably, both oral chlorhexidine and metronidazole decreased the incidence and ameliorated the severity of CIA, comparable to methotrexate. The demonstration that therapeutic eradication of *P. gingivalis* can ameliorate arthritis development [38], together with the beneficial effects of antibiotics in the human disease [39], militates in favor of the conclusion that microbial infection may somehow contribute to RA pathogenesis. In line with this view, we have pre-immunized rodents against gingipain sub-domains of *P. gingivalis* to successfully alleviate arthritis. We therefore conclude that either eradication of *P. gingivalis* [38] or pre-immunization against gingipain domains can reduce the severity of experimental arthritis, as shown here, or of oral bone loss, as shown elsewhere [37].

Previous observations suggest that a critical level of specific immune response against *P. gingivalis* can provide protection against colonization of the oral cavity by this bacterium. In periodontal patients, in situ administration of a monoclonal antibody specific for *P. gingivalis* at severely infected subgingival sites alleviated subsequent *P. gingivalis* recolonization [40], suggesting that a specific antibody has the potential to promote removal of *P. gingivalis* from the oral mucosa. In rats, immunization against *P. gingivalis* led to production of elevated levels of serum and salivary antibodies and imparted protection from oral bone loss elicited by challenge with this bacterium [41]. Consistent with these observations, our present data indicate that pre-immunization against a domain of *P. gingivalis* alleviates arthritis in the joints of experimental rats.

Accumulating evidence indicates that preclinical RA is characterized by the presence of autoantibodies in the absence of overt signs of joint inflammation and that ACPAs and rheumatoid factor (RF) can be detected up to 13 years before disease onset [42]. These disparities between early autoimmune biomarkers and joint involvement in at-risk RA subjects suggest that the inciting events that give rise to the disease may take place outside the joints [43], and it has been proposed that events occurring initially at mucosal sites play chief roles in the initial triggering stages of RA [44, 45]. First, the mucosal interface is a site of multiple host interactions with the environment. Second, in addition to the epithelial cell barrier, components of the immune system (neutrophils, macrophages, complement components, and antibodies) represent a first line of defense against environmental threats. Third, immunological events taking place at the mucosal level, i.e., specific T and B cell expansions, isotype switching, and local antibody production, are key for generation of local immune responses through migration of dendritic cells to the local lymph nodes, and subsequent induction of systemic immune responses. Fourth, the co-existence of photobionts and pathogenic microorganisms at mucosal sites likely shapes the immune repertoire and impacts both the innate and adaptive immune arms [46]. Consistently, disruption of the normal microbiota, i.e., dysbiosis, has been found to be associated with inflammatory and autoimmune conditions, including RA. It is conceivable that, under normal conditions, pathobionts, such as *P. gingivalis*, are present symbiotically at mucosal sites without negatively impacting the host, as is the case for pathogenic microbes [47]. However, under inflammatory episodes, pathobionts could trigger pro-inflammatory responses and exacerbate disease processes. This scenario is illustrated by studies of inflammatory bowel disease where the pathobiont *Helicobacter bilis* was shown to cause severe disease in the presence of a given microbiota [48]. The fact that this pathobiont is able to precipitate severe inflammatory disease without marked increases in its own abundance, without significantly altering the gut microbiota, and without being the primary target of a Th17 inflammatory response [48] is a strong indication that pathobionts can synergistically interact with microbiota to instigate or exacerbate pathology.

Recent investigations into mechanisms that underly systemic autoimmunity development demonstrated that, as a result of gut barrier breakdown, the Gram-positive gut pathobiont *Enterococcus gallinarum* can translocate into systemic organs, including the spleen, lymph nodes, and liver, and trigger a lupus-like disease in autoimmune-prone hosts [49]. In studies of neurodegenerative diseases, *P. gingivalis* and gingipains were detected in the brain of patients with Alzheimer’s disease. In an experimental model, *P. gingivalis* infection led to brain colonization and increased production of a component of amyloid plaques [50]. In that study, gingipain inhibition reduced production of amyloid plaques and reduced neuroinflammation in the treated mice. Thus, targeting gingipains either by pre-immunization, as shown in the present study, or by small-molecule inhibitors [50] has the potential to modify expression of diseases affecting the bone or the brain.

We would like to suggest that like other pathobionts [51], *P. gingivalis* is able to make use of virulence strategies found among conventional pathogens, including breaching barrier function, invading the mucosal barrier, provoking local inflammatory responses, and subverting cell signaling pathways [52]. It is possible that this pathobiont has developed multiple strategies to contribute to human disorders, including PD, RA, lupus, and Alzheimer’s disease. This view can account for the observations that *P. gingivalis* has the potential to instigate pathology locally in the oral mucosa, or remotely in the joint or in the brain. In addition to being able to act directly on colonized tissues, such as the synovium, and trigger production of specific autoantigens, inflammatory cytokines, or other factors able to promote autoimmune reactions, translocating pathobionts also could skew T cell differentiation by impacting regulatory T cells that selectively affect the activity of pro-inflammatory Th17 cells, as recently shown for *Helicobacter hepaticus* [53].

If, as the *H. bilis* findings suggest [48], multiple species of a pathobiont can act in a synergistic fashion with resident bacterial symbionts to trigger or precipitate disease, the additional putative pathobionts should be identified in RA. Future studies also should focus on gaining insight into the mechanisms through which these strategies manifest in the human disease.

## Conclusions

Pathobionts can translocate to remote tissues and instigate pathology. We have found that pre-immunization against a cysteine protease from the pathobiont *Porphyromonas gingivalis* reduces disease severity in an experimental arthritis model. We propose that specific targeting of pathobionts could alleviate pathology in chronic inflammatory diseases, such as rheumatoid arthritis.

## Supplementary information

**Additional file 1 : Figure S1**. legend. Successful expression of the gingipain proteins used.

**Additional file 2 : Figure S2.** legend. Sera from patients with RA (*n* = 155), PD (*n* = 48) or SLE (*n* = 20) were tested for reactivity with recombinant proteins HA1 (***A***), HA2/3 (***B***) HA4 (***C***) and CD (***D***) by ELISA. Healthy subjects (*n* = 35) were used as controls. Antibodies were tested for reactivity with recombinant proteins corresponding to the HA1 (A), HA2/3 (B) HA4 (C) and CD (D) by ELISA. For comparison, the Marm-Whitney test with two-sided *p* value was used. *p < 0.05, **p < 0.01, ***p < 0.001, ****p < 0.0001.

**Additional file 3 : Figure S3.** legend. Western blot analysis of the reactivity of RA and PD sera with recombinant gingipain fragments. Dilutions of serum samples from RA patients and PD patients were tested for binding with recombinant gingipain domains CD (***A***), HA1 (***B***) and HA4 (***C***). Shown are the reactivities of sera from five patient with RA and from five PD patients. Sera from five normal subjects were also used as controls. Sera were tested at a dilution of 1/100 and binding was revealed using a labeled anti-human IgG antibody diluted 1/200.

**Additional file 4 : Figure S4.** legend. Sera from patients with RA (n = 155), PD (n = 48) or SLE (n = 20) were tested for reactivity with recombinant proteins HA1 (***A***), HA2/3 (***B***) HA4 (***C***) and CD (***D***) by ELISA. Healthy subjects (n = 35) were used as controls. For comparison, the Marm-Whitney test with two-sided p value was used. *p < 0.05, **p < 0.01, ***p < 0.001, ****p < 0.0001.

**Additional file 5 : Figure S5.** legend. Lack of correlation between antibody levels to the gingipain domains HA1 (***A*** and ***E***), HA2/3 (***B*** and ***F***), HA4 (***C*** and ***G***) and CD (***D*** and ***H***) with disease activity of the RA patients tested. The Spearman correlation test was used.

## Data Availability

The datasets used and/or analyzed during the current study are available from the corresponding author, who has the ORCID identifier 0000-0002-8225-456X, on reasonable request.

## Abbreviations

CD: Arg-specific catalytic domain
CIA: Collagen-induced arthritis
HD: Hemagglutinin domain
Kgp: Lysine-specific gingipain
PD: Periodontitis
RA: Rheumatoid arthritis
RgpA: Arginine-specific gingipain A
RgpB: Arginine-specific gingipain B
