# Supplementary figures and images for: Targeting a cysteine protease from a pathobiont alleviates experimental arthritis
Source: Arthritis Res Ther. 2020 May 14;22:114. doi: 10.1186/s13075-020-02205-z (PMC7222327; doi:10.1186/s13075-020-02205-z)

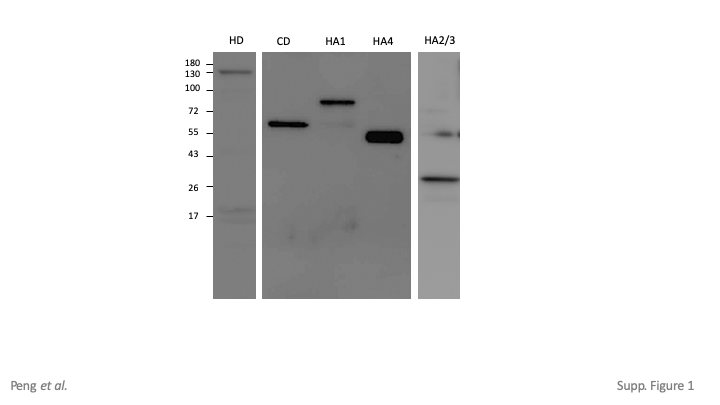

Supplement: Supplementary file 1 — Additional file 1 : Figure S1. legend. Successful expression of the gingipain proteins used. [file 13075_2020_2205_MOESM1_ESM.tiff]

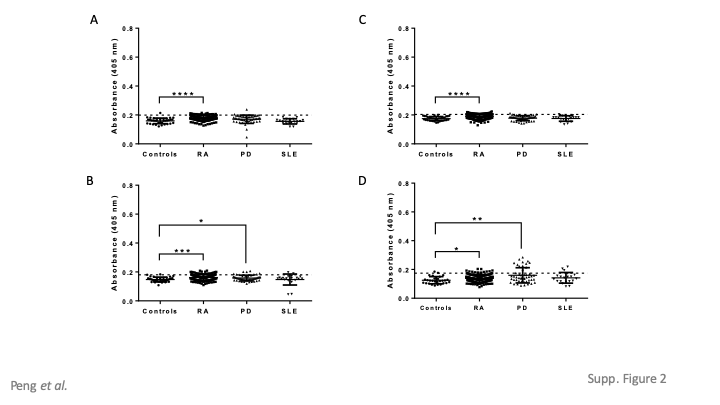

Supplement: Supplementary file 2 — Additional file 2 : Figure S2. legend. Sera from patients with RA (n = 155), PD (n = 48) or SLE (n = 20) were tested for reactivity with recombinant proteins HA1 (A), HA2/3 (B) HA4 (C) and CD (D) by ELISA. Healthy subjects (n = 35) were used as controls. Antibodies were tested for reactivity with recombinant proteins corresponding to the HA1 (A), HA2/3 (B) HA4 (C) and CD (D) by ELISA. For comparison, the Marm-Whitney test with two-sided p value was used. *p < 0.05, **p < 0.01, ***p < 0.001, ****p < 0.0001. [file 13075_2020_2205_MOESM2_ESM.tiff]

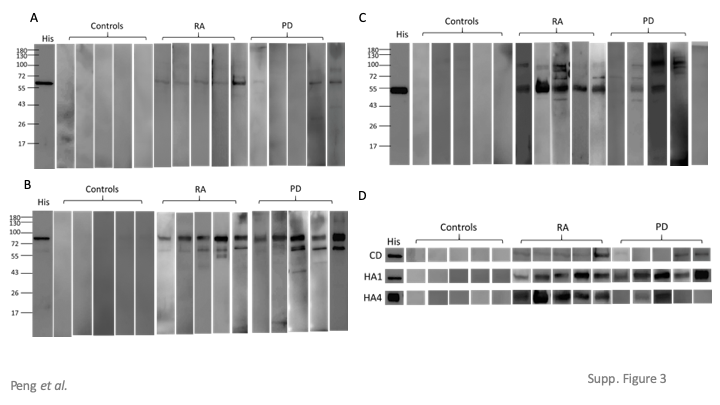

Supplement: Supplementary file 3 — Additional file 3 : Figure S3. legend. Western blot analysis of the reactivity of RA and PD sera with recombinant gingipain fragments. Dilutions of serum samples from RA patients and PD patients were tested for binding with recombinant gingipain domains CD (A), HA1 (B) and HA4 (C). Shown are the reactivities of sera from five patient with RA and from five PD patients. Sera from five normal subjects were also used as controls. Sera were tested at a dilution of 1/100 and binding was revealed using a labeled anti-human IgG antibody diluted 1/200. [file 13075_2020_2205_MOESM3_ESM.tiff]

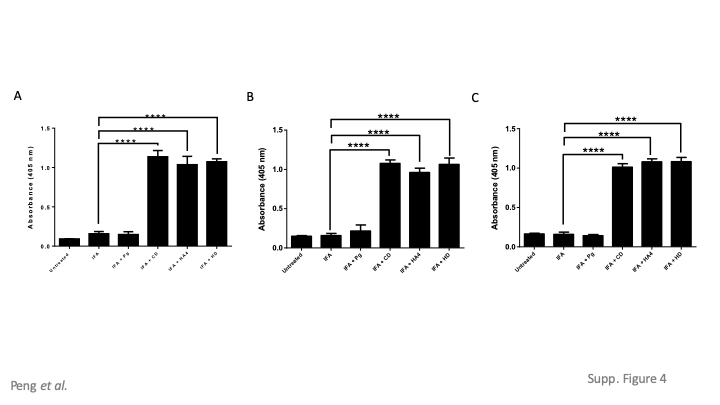

Supplement: Supplementary file 4 — Additional file 4 : Figure S4. legend. Sera from patients with RA (n = 155), PD (n = 48) or SLE (n = 20) were tested for reactivity with recombinant proteins HA1 (A), HA2/3 (B) HA4 (C) and CD (D) by ELISA. Healthy subjects (n = 35) were used as controls. For comparison, the Marm-Whitney test with two-sided p value was used. *p < 0.05, **p < 0.01, ***p < 0.001, ****p < 0.0001. [file 13075_2020_2205_MOESM4_ESM.tiff]

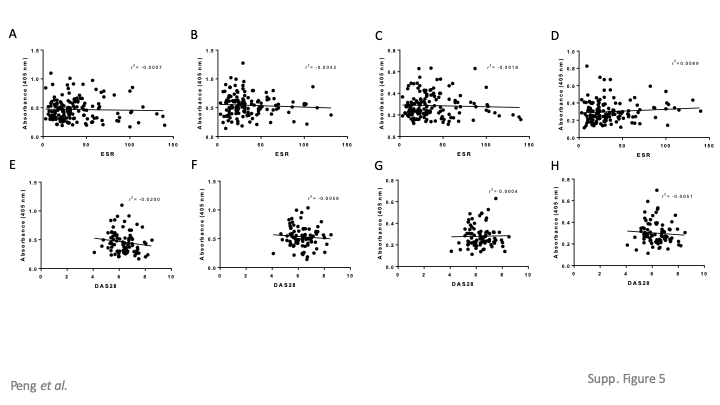

Supplement: Supplementary file 5 — Additional file 5 : Figure S5. legend. Lack of correlation between antibody levels to the gingipain domains HA1 (A and E), HA2/3 (B and F), HA4 (C and G) and CD (D and H) with disease activity of the RA patients tested. The Spearman correlation test was used. [file 13075_2020_2205_MOESM5_ESM.tiff]
